# Prognostic values of the SYNTAX score II and the erythrocyte sedimentation rate on long-term clinical outcomes in STEMI patients with multivessel disease: a retrospective cohort study

**DOI:** 10.1186/s12872-020-01490-5

**Published:** 2020-05-06

**Authors:** Chuang Li, Qian Zhang, Qianhui Wang, Jiuchang Zhong, Lefeng Wang, Kuibao Li, Xinchun Yang

**Affiliations:** Heart Center and Beijing Key Laboratory of Hypertension, Beijing Chaoyang Hospital, Capital Medical University, 8# Gong-Ti South Road, Beijing, 10020 China

**Keywords:** ST-segment elevated myocardial infarction, Major adverse cardiovascular events, Erythrocyte sedimentation rate, Multivessel coronary disease, Inflammation marker

## Abstract

**Background:**

There is a paucity of evidence on the combination of the SYNTAX score II (SSII) and erythrocyte sedimentation rate (ESR) in assessing the long-term prognosis of patients with ST-elevated myocardial infarction (STEMI) and multivessel disease. The objective of this study was to investigate whether the ESR could enhance the predictive value of SSII on the long-term prognosis of STEMI patients.

**Methods:**

A retrospective cohort study involving 483 STEMI and multivessel disease subjects receiving primary percutaneous coronary intervention was conducted. Major adverse cardiovascular events (MACE) included cardiovascular death, acute heart failure, recurrent myocardial infarction, revascularization, and nonfatal stroke. The predicted values of different models were estimated by a likelihood ratio test, Akaike’s information criteria (AIC), receiver operating characteristic (ROC) curves, net reclassification improvement (NRI), and integrated discrimination improvement (IDI).

**Results:**

During the follow-up period of up to 52 months, both the SSII and ESR were independently associated with MACE (hazard ratio [HR] = 1.032, *p* < 0.001; and HR = 1.021, *p* < 0.001, respectively). The likelihood test indicated that ESR could improve the prognostic model containing SSII (*p* < 0.001), while the combined model of SSII and ESR attained a lower AIC (*p* < 0.001). The area under the ROC curve of the combined model containing SSII and ESR increased by 0.05 (*p* = 0.04) compared to that of the model with SSII alone. The net reclassification and integrated discrimination of the SSII alone model improved significantly with ESR (NRI = 0.0319, *p* < 0.001; IDI = 0.0334, *p* < 0.001).

**Conclusions:**

The prognostic model containing SSII, which is an independent risk factor of MACE, had a significantly enhanced predictive probability with the addition of ESR.

## Background

Based on the SYNTAX score (Synergy between PCI with Taxus and Cardiac Surgery, SS) risk system, the SYNTAX score II (SSII), which integrates coronary anatomical characteristics with seven clinical risk factors, is widely recommended for identifying patients with a high risk of adverse clinical outcomes among a population with multivessel or left main coronary artery disease (CAD) and to help guide decision-making in CAD [1]. Multiple recent studies have indicated that SSII is an independent and powerful predictor of major adverse cardiovascular events (MACE), heart failure (HF), and mortality among acute coronary syndrome (ACS) patients undergoing primary percutaneous coronary intervention (PCI) and is even superior to the anatomical SYNTAX and GRACE risk scores (Global Registry of Acute Coronary Event) [2–4]. However, several studies have shown that the SSII system is not yet the optimal risk score when compared with other clinical risk scores [4, 5]. Given the results from previous studies that the combination of clinical and angiographic variables could provide more reliable predictive accuracy for clinical outcomes in ACS or multivessel PCI patients, it is necessary to incorporate other clinical parameters to the SSII system to enhance the prognostic value and further reclassify the risk of ST-elevated myocardial infarction (STEMI) patients with multivessel disease [6–9].

The erythrocyte sedimentation rate (ESR) is standardized, accurate, and generally available and reflects the plasma concentration of acute response proteins, referred to as a compound measure of both immunoglobulins and inflammation. Although several clinical studies confirmed that ESR levels were positively correlated with the progression of atherosclerosis and increased risk of coronary heart disease mortality [10–12], these findings have not yet been corroborated in patients admitted for STEMI and multivessel diseases. To date, there is only one study indicating an independent association of ESR with the prognosis of STEMI [13]. Given the above considerations, the objective of this study was to investigate the correlation of the incremental prognostic value of ESR in addition to the SSII risk system on long-term adverse clinical events among patients admitted for STEMI and multivessel disease.

## Methods

### Study population

A total of 1574 consecutive patients with a confirmed diagnosis of STEMI undergoing primary PCI in Beijing Chaoyang Hospital Heart Center between January 2014 and June 2017 were retrospectively enrolled. The diagnostic criteria of STEMI were in correspondence with the third universal definition of myocardial infarction (MI) [14]: (1) a significant increase or decrease in cardiac biomarkers,(2) persistent ischemic symptoms (within 12 h of presentation), and(3) a newly developed left bundle branch block pattern, or a new ST elevation in two or more contiguous leads, with readings of at least 0.2 mV in leads V1, V2, and V3 or at least 0.1 mV in the remaining leads. Exclusion criteria were a life expectancy of less than 6 months, a history of coronary artery bypass, or conditions resulting in a severe impairment of quality of life.

### Data collection, coronary angiography, and SYNTAX score II calculation

All the general clinical information, demographics, and treatment records were retrospectively collected from the medical database of Beijing Chaoyang Hospital. The potential inflammation status that complicated the condition of STEMI patients, including a history of chronic obstructive pulmonary disease (COPD), asthma, rheumatoid arthritis, gout, psoriasis, chronic kidney disease, and obstructive sleep apnea hypopnea syndrome (OSAHS), was identified. Laboratory tests, including white blood cell count, hemoglobin, platelet count, low-density lipoprotein, triglyceride, creatinine, fast glucose, brain natriuretic peptide (BNP), and high-sensitivity C-reactive protein (hs-CRP), were recorded within 12 h after admission. The cardiac troponin I (CTNI) and creatine kinase–myocardial band (CKMB) were consecutively censored with an interval of 6 h until the peak was obtained. The ESR was tested within 12 h after PCI by the instruments (Monitor 100, Italy), which is in accordance with the standard Westergren method. Echocardiography was also performed within 12 h postoperatively and left ventricular ejection fraction (LVEF) was recorded.

In this study, coronary angiography was reviewed and assessed by two specialists according to standards. Both investigators were blinded to the outcomes and management of medication during follow-up. Coronary single vessel disease was defined as vessel stenosis > 50% in a major coronary artery (e.g., left anterior descending coronary artery, left circumflex coronary artery, or right coronary artery) and/or in its main branches. Multiple vessel disease was defined as stenosis > 50% in at least two major coronary arteries.

Subsequently, the SYNTAX score was measured from the initial diagnostic angiogram according to the online SS calculator (www.syntaxscore.com). All lesions ≥1.5 mm in diameter and stenosis ≥50% were scored and the completely occluded infarct-related artery with TIMI (Thrombolysis in Myocardial Infarction) flow less than 1 was scored as a total occlusion within 3 months. Due to the SYNTAX score available for the stable CAD, patients with history of CABG (coronary artery bypass grafting) were excluded. Subsequently, SSII was calculated on two anatomical variables (SS and left main CAD) and six clinical variables (age, gender, COPD, peripheral arterial disease, creatinine clearance, and LVEF) using an automatic online calculation system.

### Follow-up and clinical endpoint

All subjects were followed up by telephone contact or scheduled outpatient visits to track disease development and occurrence of major clinical adverse cardiovascular events. The duration of follow-up for all patients was up to 52 months. The MACE was set as the primary endpoint of this study, which was mainly defined as a combination of cardiovascular death, rehospitalization for heart failure, recurrent nonfatal MI, repeated coronary revascularization, and nonfatal stroke.

This study was approved by the institutional review board of Beijing Chaoyang Hospital and performed in accordance with the ethical standards laid down in the 1964 Declaration of Helsinki and its later amendments. Written informed consent forms were obtained from all patients or their legal relatives.

### Statistical analysis

The normality of data distribution was tested by Shapiro–Wilk test analysis. Continuous variables were presented as mean ± standard deviation (mean ± SD) or median (interquartile range) and categorical variables as frequency (percentage). Student *t*-test and one-way analysis of variance (ANOVA) test were measured as appropriate for parametric data. Mann–Whitney *U* or Kruskal–Wallis nonparametric tests were performed on nonnormally distributed variables. The Pearson χ^2^ test or Fisher’s exact test was used to determine the differences of categorical variables, as appropriate. The Kaplan–Meier survival curves were applied to assess the incidence of MACE and the log-rank test was used to determine the intergroup differences. Univariate and multivariate Cox proportional hazard regression analyses were performed to identify predictors for adverse clinical outcomes. To evaluate the incremental prognostic value of the addition of ESR to the SSII risk system, several analytical approaches were applied to compare the changes when ESR was added in the study: (i) The receiver operating characteristic (ROC) curves were performed to assess the predictive value of SSII alone and in combination with ESR, respectively, and (ii) in order to analyze the degree to which the addition of ESR improved the predictive ability of the SSII model, the net reclassification improvement (NRI) and integrated discrimination improvement (IDI) were performed [15]. NRI is fit for the models reconstructed for participants negative or positive to events and quantifies the correct movement of categories – upwards for events and downwards for nonevents. Therefore, the event NRI (NRIe) was set as the net percentage of persons with the event of interest correctly assigned a higher predicted risk, while nonevent NRI (NRIne) as the net percentage of persons without the event of interest correctly assigned a lower predicted risk. Conversely, IDI is fit for the difference between average sensitivity and “1-specificity” for models with or without ESR and measures enhancement in average sensitivity without sacrificing the average specificity of the new model [15].(iii) A nested model was constructed to test whether a model combining the two factors could offer a better prognostic value by using the likelihood ratio test. Akaike’s information criteria (AIC) evaluated the probability that a given model is the “best” fitting model of those studied: a lower value of AIC often indicates a better fit [16]. Statistical analyses were performed using STATA (version 15.0). All statistical tests were two-tailed, and a probability value of ≤0.05 was considered statistically significant.

## Results

### Clinical information at baseline and incidence of MACE

After screening all angiographies, there were 485 STEMI and multivessel disease patients without CABG. According to the exclusion criteria, a total of 483 consecutive patients were finally enrolled and completed the follow-up in this study. The patients were divided into two groups according to the level of ESR (normal group, ESR ≤15 mm/h; and elevated group, ESR > 15 mm/h) and three groups in line with the tertiles of SSII (low SSII group, score ≤ 22; moderate SSII group, score between 22 and 33; and high SSII group, score ≥ 33) at baseline. A summary of the measurements is presented in Table 1. Patients in the elevated ESR group were older and mostly female; presented with lower body mass index (BMI), lower counts of white blood cells and platelets, lower hemoglobin, and higher hs-CRP level; and complicated with a higher prevalence of a prior history of smoking, hypertension, diabetes mellitus, COPD, and peripheral vascular disease (PVD). The elevated ESR group presented with higher SSII than that in the normal ESR group (*p* < 0.001), whereas the SYNTAX score did not show any difference. No significant difference was found in the inflammatory status, expect for a higher prevalence of chronic kidney disease among the elevated ESR group (*p* < 0.001).

**Table 1:** Baseline clinical characteristics according to ESR

	Overall	Normal group (ESR ≤ 15 mm/h)	Elevated group (ESR > 15 mm/h)	***P-value***
Number	483	359	124	
**Demographic data**				
Age (years) **	63.1 ± 12.61	61.9 ± 12.50	67.0 ± 12.14	< 0.001
Male (%) **	374 (77.4%)	301 (83.8%)	73 (58.9%)	< 0.001
BMI (kg/m^2^)	25.2 (23.2, 27.7)	25.4 (23.4, 28.7)	24.9 (22.8, 26.7)	0.04
Heart rate (beats/min)	78.1 ± 15.36	77.8 ± 14.65	78.8 ± 17.29	0.57
Systolic pressure (mmHg)	123.1 ± 22.35	123.3 ± 21.66	122.6 ± 24.35	0.77
White blood cell (×10^9^/L)	10.4 ± 3.13	10.6 ± 3.09	9.9 ± 3.22	0.04
Neutrophil (%)	79.3 ± 10.77	79.8 ± 10.66	78.0 ± 11.03	0.12
HGB (g/L) **	133.9 ± 17.79	137.7 ± 16.27	122.8 ± 17.42	< 0.001
Platelet (×10^9^/L)**	208 (172, 237)	205 (170, 231)	225.5 (178, 261)	< 0.001
Cholesterol (mmol/L)	4.5 ± 1.11	4.5 ± 1.11	4.6 ± 1.11	0.36
HDL (mmol/L)	1.1 ± 0.29	1.1 ± 0.30	1.0 ± 0.27	0.12
LDL (mmol/L)	2.8 ± 0.94	2.8 ± 0.93	2.9 ± 0.95	0.30
Triglyceride (mmol/L)	1.7 ± 1.47	1.7 ± 1.58	1.6 ± 1.11	0.41
hs-CRP (mg/L) *	5.4 (2.29, 11.13)	4.4 (2.06, 10.16)	11.0 (4.37, 13.06)	< 0.01
Creatinine (μmol/L)	76.4 (65.4, 93.6)	76.4 (65.6, 88.4)	76.3 (64.8, 106.1)	0.18
Fasting glucose (mmol/L)	8.3 ± 3.97	8.0 ± 3.80	8.9 ± 4.38	0.05
BNP (pg/ml) *	127.0 (45.4, 382.0)	93.7(39.6, 271.0)	255 (123.0, 656)	< 0.01
CTNI (ng/mL)	30.1 (9.21, 77.39)	32.0 (10.74,79.8)	25.2 (5.44, 69.46)	0.08
EF (%)	60 (50,67)	60 (51, 68)	59 (48.5,65)	0.05
**Risk factors**				
Hypertension (%) *	290 (60.0%)	259 (55.7%)	90 (72.6%)	< 0.01
Diabetes mellitus (%)	175(36.2%)	119 (33.1%)	56 (45.2%)	0.02
Hyperlipidemia (%)	138 (28.8%)	358 (29.3%)	33 (27.1%)	0.63
Smoking (%) * *	295 (61.1%)	238 (66.3%)	57 (46.0%)	< 0.001
Previous PCI (%)	45 (9.3%)	36 (10.0%)	9 (7.2%)	0.47
Previous MI (%)	59 (12.2%)	45 (12.6%)	14 (11.3%)	0.78
COPD (%) *	16 (3.3%)	7 (2.0%)	9 (7.3%)	< 0.01
PVD (%)	54 (11.2%)	34 (9.5%)	20 (16.1%)	0.04
Asthma (%)	2 (0.4%)	2(0.6%)	0(0.0%)	0.41
Rheumatoid arthritis (%)	3 (0.62%)	1(0.3%)	2 (1.6%)	0.10
Gout (%)	8 (1.7%)	7 (2.0%)	1 (0.8%)	0.39
Psoriasis (%)	2 (0.4%)	1 (0.3%)	1 (0.8%)	0.43
OSAHS (%)	6 (1.0%)	5 (1.4%)	0 (0.0%)	0.19
Chronic kidney disease (%) * *	17(3.5%)	6 (1.7%)	11 (8.9%)	< 0.001
**Infarct-related artery**				
Left main artery (%) *	5(1.0%)	5 (1.4%)	0 (0%)	< 0.01
Left anterior descending artery (%)	193 (40.0%)	136(37.9%)	57 (46.0%)	0.16
Left circumflex artery (%)	74(15.3%)	58 (16.2%)	16 (12.9%)	0.39
Right coronary artery (%)	211(43.7%)	160 (44.6%)	51 (41.1%)	0.51
SYNTAX score	26.5 ± 9.61	26.6 ± 9.96	26.3 ± 8.56	0.74
SSII* *	32.3 ± 11.34	30.6 ± 10.98	37.1 ± 11.04	< 0.001
**MACE**				
Follow-up time (months) *	25 (20, 35)	27 (21, 36)	22 (10.5, 30)	< 0.01
Mortality (%)	28 (5.8%)	16 (4.5%)	12 (9.7%)	0.03
Heart failure (%) **	43 (8.9%)	16 (4.5%)	27 (21.8%)	< 0.001
Recurrent MI (%)	21 (4.4%)	18 (5.0%)	3 (2.4%)	0.22
Revascularization (%)	65 (13.5%)	41(11.4%)	24 (19.4%)	0.03
Nonfatal stroke (%)	6 (1.2%)	2(0.6%)	4 (1.6%)	0.04
MACE (%) **	139 (27.8%)	82 (22.8%)	57 (46.0%)	< 0.001

The values are expressed as mean ± standard deviation, median (IQR, observations available) or number (percentage). Abbreviations: *BMI* body mass index, *HGB* hemoglobin, *HDL* high-density lipoprotein, *LDL* low-density lipoprotein, *hs-CRP* high-sensitivity C-reactive protein, *ESR* erythrocyte sedimentation rate, *BNP* brain natriuretic peptide, *EF* ejection fraction, *CTNI* cardiac troponin I, *COPD* chronic obstructive pulmonary disease, *PVD* peripheral vascular disease, *OSAHS* obstructive sleep apnea hypopnea syndrome, *SSII* SYNTAX score II, *MACE* major adverse cardiovascular events, *MI* myocardial infarction. * *p* < 0.01; * * *p* < 0.001

**Fig. 1:**
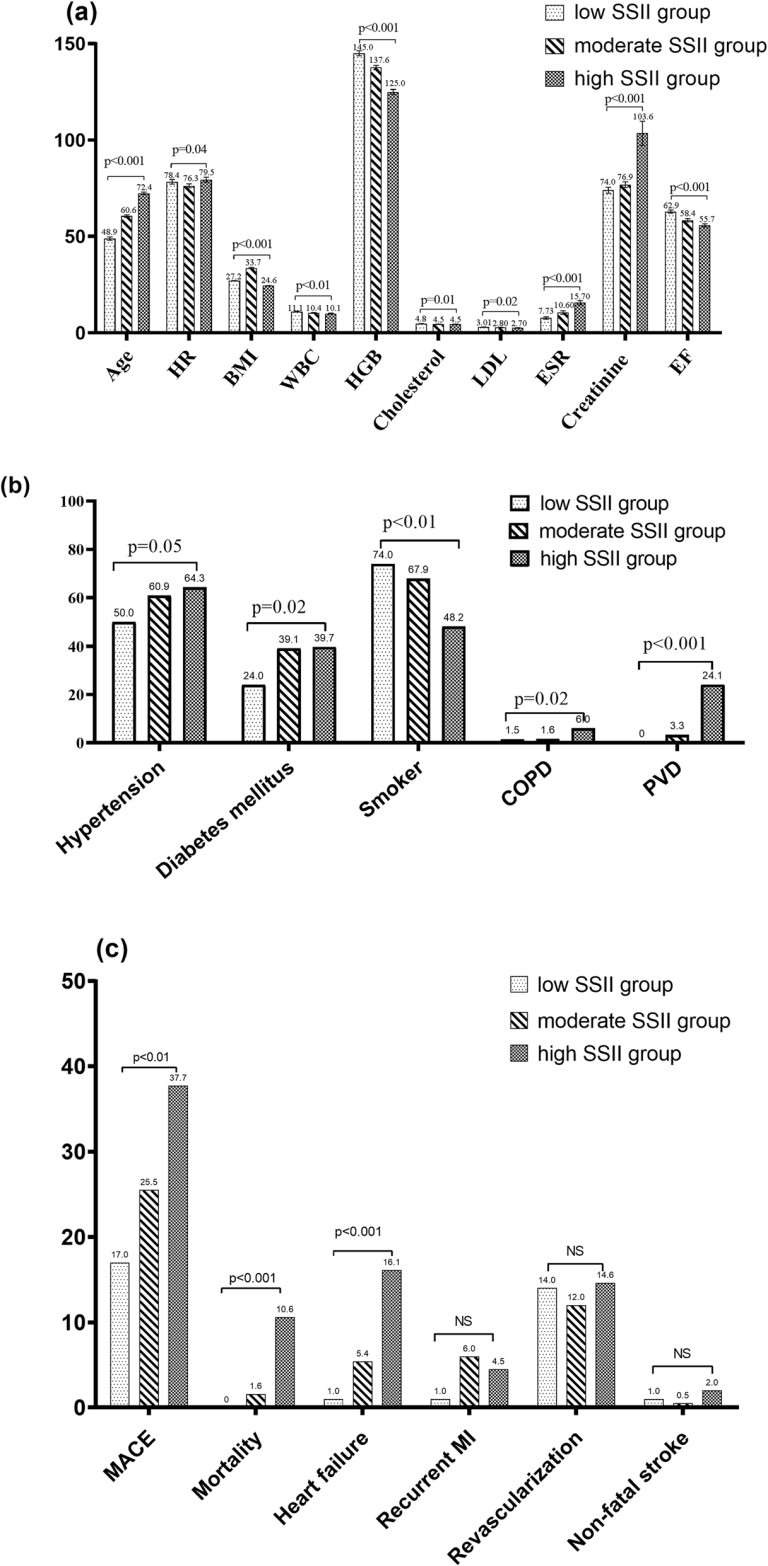
Comparison of baseline values among the three SSII groups. **a** Distribution of baseline and laboratory tests, the values are reported as the mean ± SD; **b** distribution of cardiovascular risk factors; **c** distribution of major adverse cardiovascular events (MACE) including morality, heart failure, recurrent myocardial infarction (MI), revascularization, and nonfatal stroke. Abbreviations: HR, heart rate on admission; BMI, body mass index; WBC, white blood cell counts; HGB, hemoglobin; LDL, low-density lipoprotein; ESR, erythrocyte sedimentation rate; EF, ejection fraction; COPD, chronic obstructive pulmonary disease; PVD, peripheral vessel disease; SD, standard deviation

As is usually reported in similar cohorts of patients, the average age, BMI, admission heart rate, white blood cell counts, and creatinine were significantly different among the three SSII groups. Patients in the high SSII group had a higher prevalence of diabetes mellitus, hypertension, smoking, COPD, and PVD than the prevalence in those in the low SSII group (Fig. 1 and Supplementary Table 1). Compared with the low SSII group, lower LVEF (p < 0.001), higher ESR level (p < 0.001), and lower levels of cholesterol (*p* = 0.01) and low-density lipoprotein (*p* = 0.02) were significantly exhibited by the high SSII group. There were no significant differences in other characteristics or variables among the three groups.

A total of 139 (27.8%) subjects reached the clinical endpoint, including 28 (5.8%) cardiovascular deaths, 21 (4.4%) recurrent MIs, 65 (13.5%) repeat revascularizations, 43 (8.9%) heart failures, and 6 (1.2%) nonfatal strokes. The overall MACE was significantly higher for the elevated ESR group compared with those in the normal group (46.0% vs. 22.8%, respectively, *p* < 0.001). Patients in the elevated ESR group were prone to suffer a higher frequency of mortality (9.7% vs. 4.5%, *p* = 0.03), heart failure (21.8% vs. 4.5%, *p* < 0.001), revascularization (19.4% vs. 11.4%, p = 0.03), and nonfatal stroke (1.6% vs. 0.6%, *p* = 0.04). Additionally, when stratified by the tertiles of the SSII risk system, the incidence of MACE was significantly higher in the high SSII group (37.7% vs. 25.5% vs. 17.0%, *p* < 0.01), particularly in mortality (10.6% vs. 3.85% vs. 0.0%, *p* < 0.001) and heart failure (16.1% vs. 5.4% vs. 1.0%, *p* < 0.001), when compared with those in moderate SSII group and low SSII group, respectively.

### Regression analysis on MACE

**Fig. 2:**
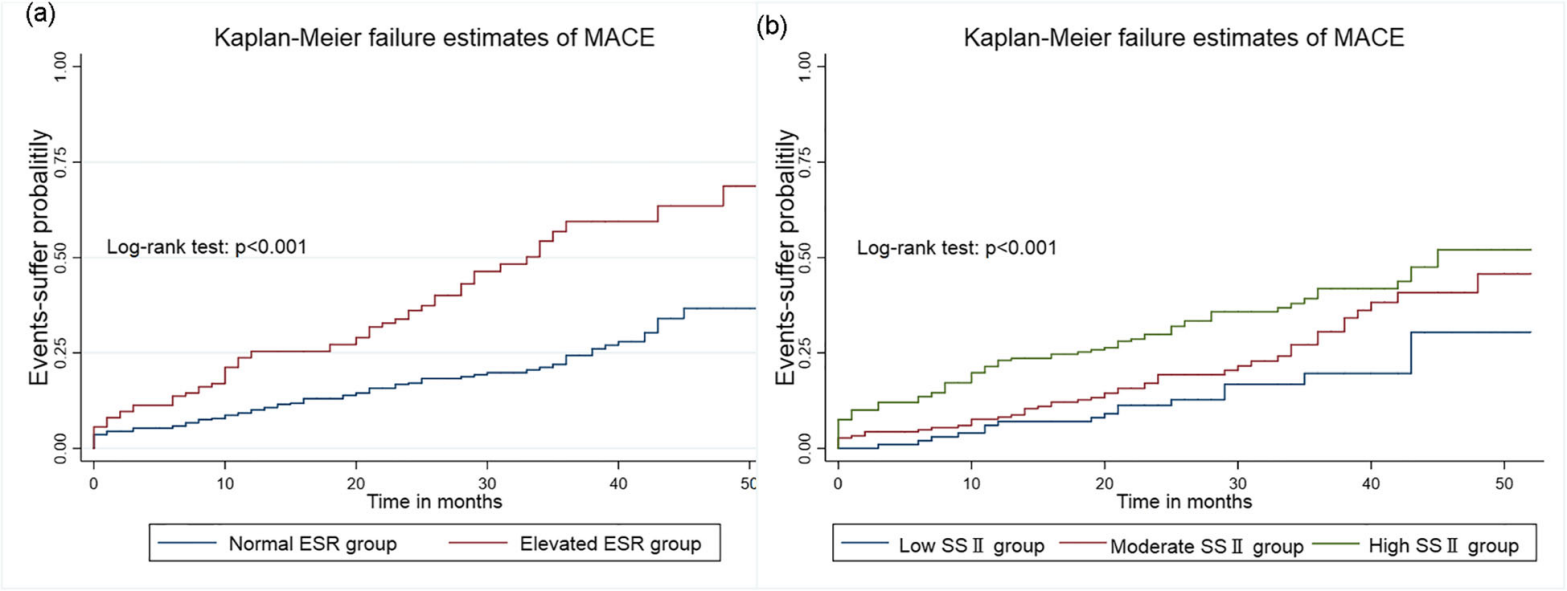
Kaplan–Meier failure curve of MACE during the follow-up period of 52 months according to the values of ESR and SYNTAX score II (SSII). Patients in the elevated ESR groups as well as those in the high SSII groups were prone to suffer MACE

Subsequently, the cumulative incidences of MACE among the two ESR groups and three SSII groups were assessed by a Kaplan–Meier survival curve (Fig. 2). It was demonstrated that patients with elevated ESR had a significantly higher occurrence of MACE compared to those in the normal level. In contrast to those with moderate and high SSII, decreased event-suffer probability was observed in the low SSII group. A log-rank test on the curve identified significant differences between the two ESR groups (χ^2^ = 31.97, *p* = 0.01) and among the three SSII groups (χ^2^ = 15.99, *p* < 0.001) in the cumulative incidence of MACE in patients with STEMI and multivessel disease.

The Cox proportional hazard regression analysis results are shown in Table 2. Univariate analysis revealed that both SSII (HR = 1.038, 95% CI = 1.024–1.053, *p* < 0.001) and ESR level (HR = 1.027, 95% CI = 1.022–1.041, *p* < 0.001) were associated with an increased risk of MACE. We took into account the multicollinearity between SSII and its continuous parameters (SS, age, LVEF, and creatinine); hence, those parameters did not enter into the multivariate regression analysis following SSII. After adjustment for other potential confounders including BMI, admission heart rate, admission systolic pressure, cholesterol, low-density lipoprotein, hs-CRP, and a history of hypertension, diabetes mellitus, hyperlipidemia, or smoking, both SSII and ESR were still independent powerful predictors for the incidence of MACE in patients with STEMI and multivessel disease (ESR, HR = 1.022, 95% CI = 1.012–1.033, *p* < 0.001; and SSII, HR = 1.033, 95% CI = 1.017–1.049, *p* < 0.001).

**Table 2:** Cox proportional hazard regression analyses for major adverse cardiovascular events

	Univariate analysis		Multivariable analysis	
	HR (95%CI)	P	HR (95%CI)	P
Age	1.021 (1.007, 1.036)	< 0.01		
Male	1.528 (1.051, 2.22)	0.03		
BMI	0.998 (0.994, 1.005)	0.97	1.001 (0.996, 1.007)	0.46
Heart rate	1.008 (0.997, 1.019)	0.05	1.008 (0.999, 1.018)	0.07
Systolic pressure	1.010 (1.001, 1.018)	0.03	1.008 (1.000, 1.016)	0.04
Cholesterol	1.174 (1.001, 1.368)	0.04	1.179 (0.874, 1.593)	0.28
LDL	1.139 (0.947, 1.367)	0.17	0.968 (0.666, 1.406)	0.86
ESR	1.027 (1.022, 1.041)	< 0.001	1.022 (1.012, 1.033)	< 0.001
SSII	1.038 (1.024, 1.053)	< 0.001	1.033 (1.017, 1.049)	< 0.001
hs-CRP	1.003 (1.002, 1.004)	< 0.001	1.002 (1.000, 1.003)	< 0.01
Creatinine	1.004 (1.001, 1.006)	< 0.01		
BNP	1.000 (1.000, 1.102)	< 0.01		
Fasting glucose	1.059 (1.019, 1.003)	< 0.01	1.038 (0.988, 1.089)	0.14
EF	0.978 (0.962, 0.995)	0.01		
Hypertension	1.940 (1.303, 2.888)	0.001	1.568 (1.060, 2.319)	0.02
Diabetes mellitus	1.408 (1.004, 1.975)	0.05	0.942 (0.616, 1.441)	0.78
Hyperlipidemia	1.937 (1.338, 2.806)	< 0.001	1.118 (0.766, 1.632)	0.56
Smoking	0.697 (0.485, 1.002)	0.05	1.105 (0.760, 1.607)	0.60
PVD	1.729 (1.086, 2.755)	0.02		
COPD	2.287 (1.163, 4.499)	0.02		

Abbreviations: BMI, body mass index; LDL, low-density lipoprotein; BNP, brain natriuretic peptide; EF, ejection fraction; COPD, chronic obstructive pulmonary disease; PVD, peripheral vascular disease; hs-CRP, high-sensitivity C-reactive protein; ESR, erythrocyte sedimentation rate; SSII, SYNTAX score II

### Combination of SSII with ESR in predicting clinical outcomes

By using the likelihood ratio, the nested models were used to classify which one could provide better positive predicted probability on different clinical outcomes. The combined model provided a better fit with a lower AIC than that in models containing SSII alone (MACE, 1284.235 vs. 1299.304, *p* < 0.001; cardiovascular death, 295.6914 vs. 299.6875, *p* < 0.01; heart failure, 438.9405 vs. 461.0, *p* < 0.001) (Table 3).

**Table 3:** Akaike’s information criteria and likelihood ratio test to determine the best fitting model for predicting MACE, cardiovascular death, and heart failure

		Akaike’s information criteria	Likelihood ratio test		
Clinical outcomes	Model	AICc	χ^2^	df	P-value
MACE	SSII	1299.304			
	SSII + ESR	1284.235	17.07	1	< 0.001
Cardiovascular death	SSII	299.6875			
	SSII + ESR	295.6914	6.00	1	0.01
Heart failure	SSII	461.0			
	SSII + ESR	438.9405	24.06	1	< 0.001

Abbreviations: AICc, corrected Akaike’s information criteria; *SSII* SYNTAX score II, *ESR* erythrocyte sedimentation rate, *MACE* major adverse cardiovascular events

**Fig. 3:**
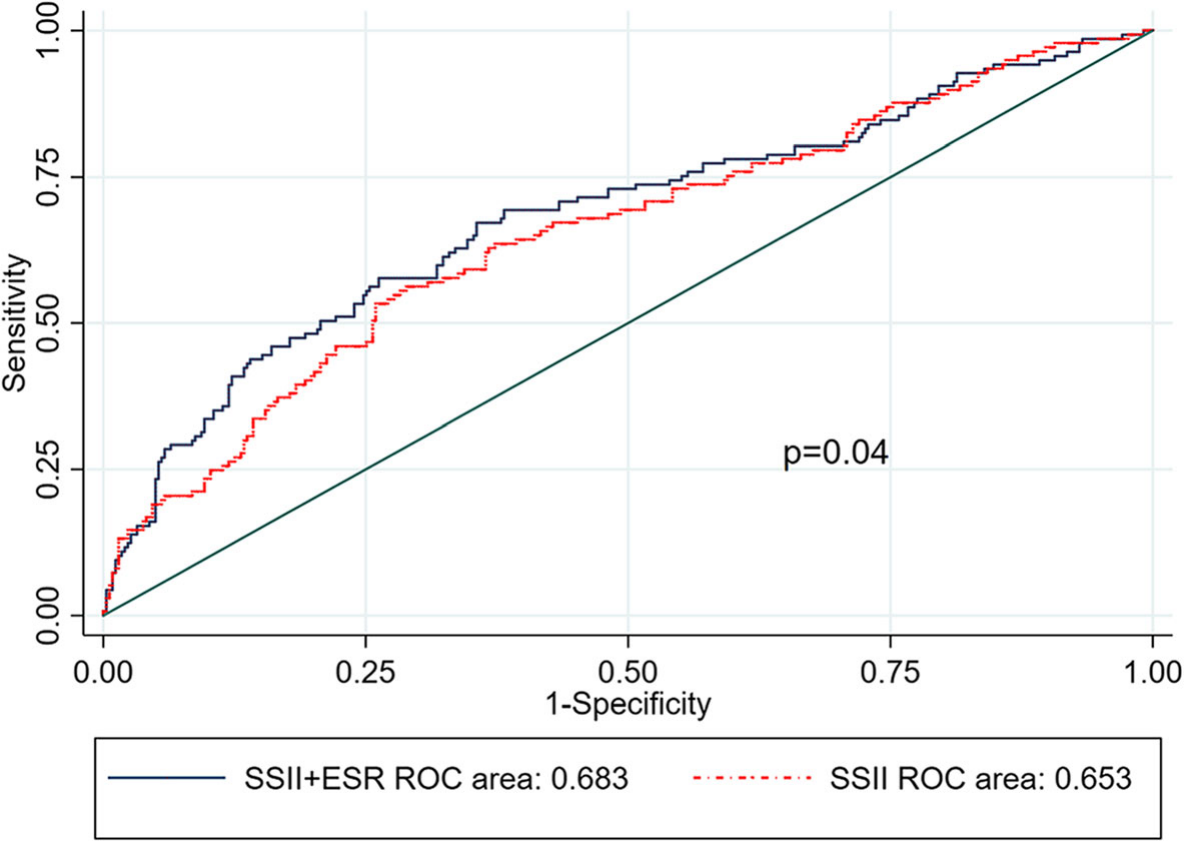
Receiver operating characteristic (ROC) curve for the combined models and SYNTAX score II (SSII) alone in predicting long-term MACE. Combined models containing SSII and ESR slightly improved the predictive value over SSII

The additive prognostic value of ESR in addition to SSII was evaluated by an increase in the area under the ROC curve (AUC) (Fig. 3 and Supplementary Figs. 1 and 2). The AUC of the combined models regarding MACE, cardiovascular death, and heart failure was 0.683, 0.8802, and 0.8467, respectively, of which all were higher than that for SSII alone (MACE, 0.653, *p* = 0.04; cardiovascular death, 0.8546, *p* = 0.22; heart failure, 0.7743, *p* < 0.01).

Models combining ESR and SSII significantly improved the net reclassification in predicting compound events and heart failure (Table 4). The combined model analysis results revealed a significant improvement in reclassification by 6.5% for those with MACE and by 38.4% for those without, with both reaching a significant statistical difference (*p* < 0.001). Therefore, the combination of SSII and ESR is likely to predict a lower risk of MACE than that in SSII alone, and subsequently provide a 31.9% improvement in net reclassification in the nonevent group. Meanwhile, in the setting of cardiovascular death, all measures of discrimination and reclassification, the combination of SSII and ESR resulted in significant improvement by 66.8 and 20.9% in the nonevent and event groups, respectively (NRIne, 0.668; NRIe, 0.209; NRI, 0.877, *p* < 0.001). IDI analysis results also indicated that the model predictive value on MACE and heart failure, but not cardiovascular death, was significantly improved by the addition of ESR to the SSII system (MACE, 0.033, *p* < 0.001; cardiovascular death, 0.120, *p* = 0.57; heart failure, 0.099, *p* < 0.01) (Table 4).

**Table 4:** Net reclassification improvement for model improvement with the addition of ESR to SSII alone

SSII vs. SSII and ESR				
NRI	NRIe	NRIne	Total	*P*-value
MACE	−0.065	0.384	0.319	0.001
Cardiovascular death	−0.142	0.626	0.484	0.01
Heart failure	0.209	0.668	0.877	< 0.001
IDI	IDIe	IDIne	Total	*P*-value
MACE	0.024	−0.009	0.033	< 0.001
Cardiovascular death	0.0113	−0.0007	0.120	0.57
Heart failure	0.090	−0.009	0.099	< 0.01

Abbreviations: *NRI* net reclassification index, *IDI* integrated discrimination improvement, *SSII* SYNTAX score II, *ESR* erythrocyte sedimentation rate, *MACE* major adverse cardiovascular events

## Discussion

In this retrospectively cohort study, our findings indicate: (1) the SSII system as well as ESR are an independent predictor of long-term adverse clinical events in patients with STEMI and multivessel disease, and (2) models containing ESR and the SSII system could efficiently enhance the predicted probability of long-term cardiovascular death, heart failure, and MACE, but not of recurrent MI, repeat revascularization, or nonfatal stroke. When ESR and SSII are jointly used to assess compound adverse outcomes and heart failure in particular, the AUC of the combined prognostic model significantly increased. Moreover, calibration, discriminatory capacity, and reclassification of SSII scoring are improved significantly by the integration of ESR. The results suggest that the ESR level at admission is likely to accurately and efficiently enhance the predictive value of the SSII system for major clinical adverse cardiovascular events in patients with multivessel disease suffering STEMI.

The SSII score, which accounts for coronary anatomy and demographic and clinical factors, has been well known to be superior to the conventional SYNTAX score and is recommended in guiding decision-making on the treatment of left main CAD or complex three-vessel disease in ESC guidelines [1, 17]. Recently, a pooled analysis by Cavalcante et al. indicated that the SSII score, independent of diabetes, is a considerably powerful prediction model for long-term outcomes in patients with multivessel disease following PCI or CABG [18]. Similarly, previous studies showed that the SSII risk system is also a significant predictor of long-term mortality in patients with STEMI and is even better than other risk scoring systems including GRACE and TIMI scores [2, 4, 19, 20]. However, patients with multivessel disease in the setting of STEMI tend to increase the frequency of incomplete revascularization and serious complications and suffer the phenomenon of no flow after primary PCI [21, 22]. In the study of Onuk et al., the anatomical SYNTAX score and clinical SYNTAX score were not correlated with long-term adverse events and mortality in the PCI arm of patients with STEMI and multivessel disease [23]. It is noteworthy that the limited number of patients and retrospective nature of the study potentially contributed to the negative results. Although SSII is independently associated with long-term MACE, cardiovascular morality, and heart failure in patients with STEMI and multivessel disease, there is still room for improvement in its risk stratification and reclassification for clinical outcomes as presented in our study.

Some inflammatory markers, such as CRP, ESR, and von Willebrand factor concentration, have been generally considered as having vital roles in the initiation, progression, and complication of atherosclerosis and as independent predictors of mortality and adverse cardiovascular events [24, 25]. The ESR is a direct measure of red blood cell aggregation and has been a prognostic marker in patients suffering STEMI [12, 13]. It is well known that ESR has been found to intimately correlate with the incidence of MI and cardiac morality in the general population [10, 26]. In the study by Brunettietal, the average ESR levels in patients receiving untimely reperfusion consistently increased by nearly three times from admission to 4 days in the hospital, while the CRP level had reached a plateau within 2 days [27]. Due to the characteristic stability and sensitivity of the acute phase response, the ESR is less influenced by major inflammatory markers and nearly parallel to the complete resolution of inflammation in comparison to hs-CRP, which is prone to fluctuate following active infection or medication-taking [28, 29]. In contrast, inflammation responding to acute MI is always a rigid and serious reaction following myocardial necrosis and plaque rupture. Plaque rupture and late-stage atherosclerosis in the pathophysiology of STEMI may be presented as intimal hemorrhage. In this circumstance, erythrocyte membranes are abundant in late-stage lesions and may be a proinflammatory factor, increasing the risk of plaque rupture [30]. Hence, our hypothesis is entirely rational that an elevated ESR could be a useful prognostic factor for clinical outcomes following STEMI.

In addition, our findings provided some evidence regarding the additive values of ESR into the SSII system for the prediction of rehospitalization related to developing heart failure after efficient reperfusion by primary PCI. Heart failure has been shown to have a critical influence in patients after STEMI [31]; nevertheless, the evaluation of HF after STEMI has not been appropriately implemented in real-life cohorts. In patients with rheumatoid arthritis who developed new-onset heart failure, Maradit-Kremers et al. discovered that there was a peak in the proportion of patients with increased ESR prior to the 6-month interval before heart failure diagnosis [32]. Accordingly, we speculate that ESR signals raised the deterioration of impaired cardiac function and the prevalence of new-onset heart failure during follow-up, shedding some light on the prognosis of STEMI patients.

In our study, the finding that there was no statistical difference in repeat revascularization and recurrent MI is not the same as other studies [20], probably due to the complete revascularization and optical medication strategy in our center. Similarly, results from two large cohort studies, which enrolled 1689 and 1917 patients with STEMI indicated that the increase of reinfarction and revascularization did not reach statistical significance [4, 33]. Hence, the function of the SSII prediction model over soft ischemia events still remain unclear and needs to be elaborated.

### Limitation

In this retrospective cohort study from a single center, some limitations should be cautioned. First, due to the relatively small number of eligible patients with STEMI and multivessel disease from only one cardiac center, our findings are inevitably affected by a selection bias. Further large multicenter studies should be conducted. Second, although there is a statistical significance between the two ESR groups, a low incidence of non-fatal stroke cases probably contributed to some restrictions on the evaluation of cerebrovascular events in this study. It is less reasonable that the anatomical SYNTAX score and derivative SSII have access to the prevalence of non-stroke patients. Third, we did our best to complete the follow-up, but it is inevitable that a few admitted to other hospitals are missing because of the fluent population in Beijing. Finally, as STEMI patients accompanied with inflammation disorders were not excluded in this study, it is uncertain that the elevated inflammation marker is potentially influenced by inflammation status. However, whether the patients were complicated with other inflammation disorders, the ESR could assess the extent of inflammation activity and predict coronary atherosclerosis and cardiac mortality.

## Conclusions

According to the results from this retrospective cohort study, it is worth noting that SSII along with ESR is a strong independent predictor of adverse clinical outcomes in STEMI patients with multivessel disease after adjusting for traditional cardiovascular risk factors. Moreover, the ESR could enhance the predictability of a model containing SSII for the prognosis of patients with STEMI and multivessel disease.

## Supplementary information

**Additional file 1: Supplementary Table 1.** Baseline clinical characteristics according to the tertiles of SYNTAX score II.

**Additional file 2: Supplementary Fig. 1.** Receiver operating characteristic (ROC) curve for the combined models and SYNTAX score II (SSII) alone in predicting morality).

**Additional file 3: Supplementary Fig. 2.** Receiver operating characteristic (ROC) curve for the combined models and SYNTAX score II (SSII) alone in predicting acute heart failure.

## Data Availability

The datasets generated and analyzed during the current study are not publicly available due to the restrictions by the Beijing Chaoyang Hospital who is the data owner. The authors used this dataset under an agreement with the Beijing Chaoyang Hospital for the current study.

## Abbreviations

STEMI: ST-segment elevated myocardial infarction
ACS: Acute coronary syndrome
MACE: Major adverse cardiovascular events
HF: Heart failure
SYNTAX: Synergy between PCI with Taxus and cardiac surgery
SSII: SYNTAX score II
CAD: Coronary artery disease
PCI: Percutaneous coronary intervention
GRACE: Global registry of acute coronary event
TIMI: Thrombolysis in myocardial infarction
MI: Myocardial infarction
CABG: Coronary artery bypass grafting
ESR: Erythrocyte sedimentation rate
hs-CRP: High-sensitivity C-reactive protein
CTNI: Cardiac troponin I
CKMB: Creatine kinase–myocardial band
BMI: Body mass index
BNP: Brain natriuretic peptide
LVEF: Left ventricular ejection fraction
COPD: Chronic obstructive pulmonary disease
PVD: Peripheral vascular disease
OSAHS: Obstructive sleep apnea hypopnea syndrome
HR: Hazard ratio
CI: Confidence interval
ROC: Receiver operating characteristic curve
AUC: Area under the curve
NRI: Net reclassification improvement
IDI: Integrated discrimination improvement
AIC: Akaike’s information criteria
