# Supplementary material for: Prognostic values of the SYNTAX score II and the erythrocyte sedimentation rate on long-term clinical outcomes in STEMI patients with multivessel disease: a retrospective cohort study
Source: BMC Cardiovasc Disord. 2020 May 6;20:213. doi: 10.1186/s12872-020-01490-5 (PMC7204004; doi:10.1186/s12872-020-01490-5)
Supplement: Supplementary file 1 — Additional file 1: Supplementary Table 1. Baseline clinical characteristics according to the tertiles of SYNTAX score II. [file 12872_2020_1490_MOESM1_ESM.docx]

**Supplementary table 1****. Baseline clinical characteristics according to the tertiles of SYNTAX score Ⅱ**

|  | **Low SSⅡ group** | **Moderate SSⅡ group** | **High SSⅡ group** | **P-value** |
| --- | --- | --- | --- | --- |
| Number | 100 | 184 | 199 |  |
| Age (years)^**^ | 48.9±8.68 | 60.6±8.64 | 72.4±9.29 | <0.001 |
| Male (%)**^**^** | 99 (99.0%) | 164 (89.1%) | 111 (55.8%) | <0.001 |
| BMI (kg/m^2^)^**^ | 26.7(24.7,29.2) | 25.3(23.2,27.5) | 24.5(22.1,26.4) | <0.001 |
| Heart Rate (beats/min) | 78.4±12.05 | 76.3±13.72 | 79.5±17.95 | 0.04 |
| WBC (10^9^/L)^*^ | 11.1±2.53 | 10.4±3.22 | 10.1±3.29 | <0.01 |
| HGB (g/L)^**^ | 145.0±13.02 | 137.6±15.35 | 125.0±17.72 | <0.001 |
| Cholesterol (mmol/L) | 4.8±1.06 | 4.5±1.07 | 4.5±1.15 | 0.01 |
| LDL (mmol/L) | 3.1±0.90 | 2.8±0.93 | 2.7±0.95 | 0.02 |
| ESR (mm/h)^**^ | 7.7±6.30 | 10.6±11.96 | 15.7±15.62 | <0.001 |
| Creatinine (umol/L) ^**^ | 74.0±15.45 | 76.9±19.70 | 103.6±88.02 | <0.001 |
| Fasting Glucose(mmol/L) | 7.9±3.87 | 8.0 ±3.63 | 8.7±4.28 | 0.18 |
| EF (%) ^**^ | 62.9±8.25 | 58.4±11.98 | 55.7±11.66 | <0.001 |
| Hypertension (%) | 50(50.0%) | 112 (60.9%) | 128 (64.3%) | 0.05 |
| Diabetes mellitus (%) | 24(24.0%) | 72 (39.1%) | 79(39.7%) | 0.02 |
| Smoking (%)^*^ | 74 (74.0%) | 125 (67.9%) | 96 (48.2%) | <0.01 |
| COPD (%) | 1 (1.5%) | 3 (1.6%) | 12 (6.0%) | 0.02 |
| PVD (%) ^**^ | 0 (0.0%) | 6 (3.3%) | 48 (24.1%) | <0.001 |
| MACE(%)^**^ | 17 (17.0%) | 47 (25.5%) | 75 (37.7%) | <0.001 |
| Mortality(%)^**^ | 0 (0.0%) | 7 (3.8%) | 21 (10.6%) | <0.001 |
| Heart failure(%)^**^ | 1 (1.0%) | 10(5.4%) | 32 (16.1%) | <0.001 |
| Recurrent MI(%) | 1(1.0%) | 11 (6.0%) | 9 (4.5%) | 0.14 |
| Revascularization(%) | 14 (14.0%) | 22 (12.0%) | 29 (14.6%) | 0.74 |
| Non-fatal stroke(%) | 1 (1.0%) | 1 (0.5%) | 4 (2.0%) | 0.42 |

The values are expressed as mean ± standard deviation, median (IQR, Observations available) or number (percentage). Abbreviations: SSⅡ, SYNTAX score Ⅱ; BMI, body mass index; LDL, low density lipoprotein; ESR, erythrocyte sedimentation rate; EF, ejection fraction; COPD, chronic obstruct pulmonary disease; PVD, peripheral vascular disease; MACE, major adverse cardiovascular events; **, p<0.001; *, p<0.01.
