# Supplementary figures and images for: Prognostic values of the SYNTAX score II and the erythrocyte sedimentation rate on long-term clinical outcomes in STEMI patients with multivessel disease: a retrospective cohort study
Source: BMC Cardiovasc Disord. 2020 May 6;20:213. doi: 10.1186/s12872-020-01490-5 (PMC7204004; doi:10.1186/s12872-020-01490-5)

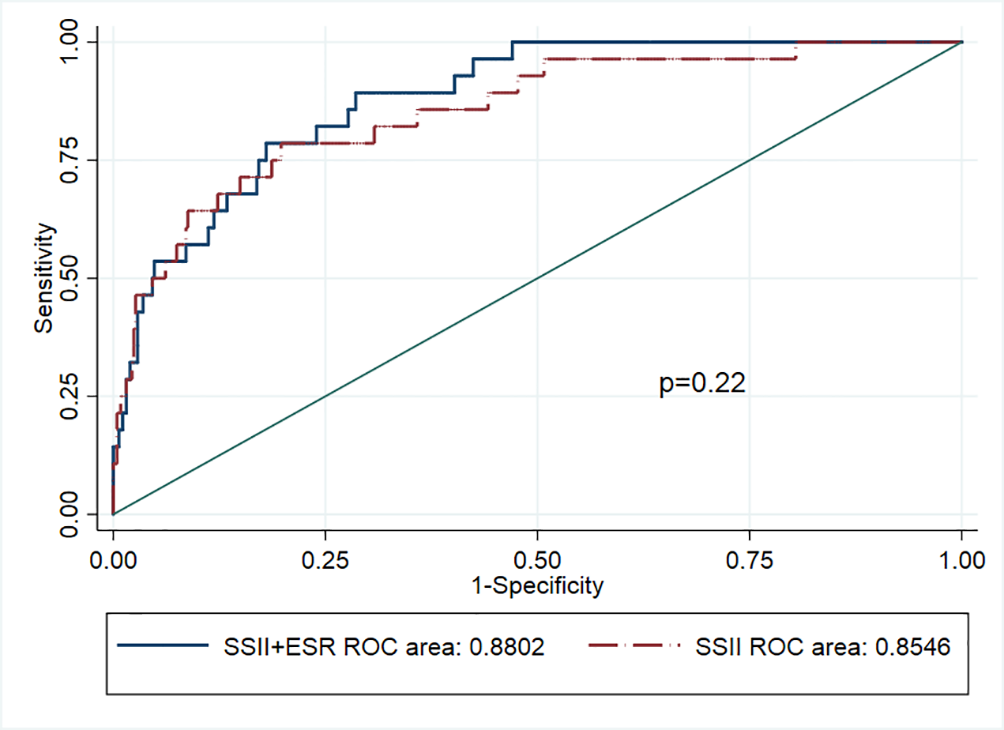

Supplement: Supplementary file 2 — Additional file 2: Supplementary Fig. 1. Receiver operating characteristic (ROC) curve for the combined models and SYNTAX score II (SSII) alone in predicting morality). [file 12872_2020_1490_MOESM2_ESM.tif]

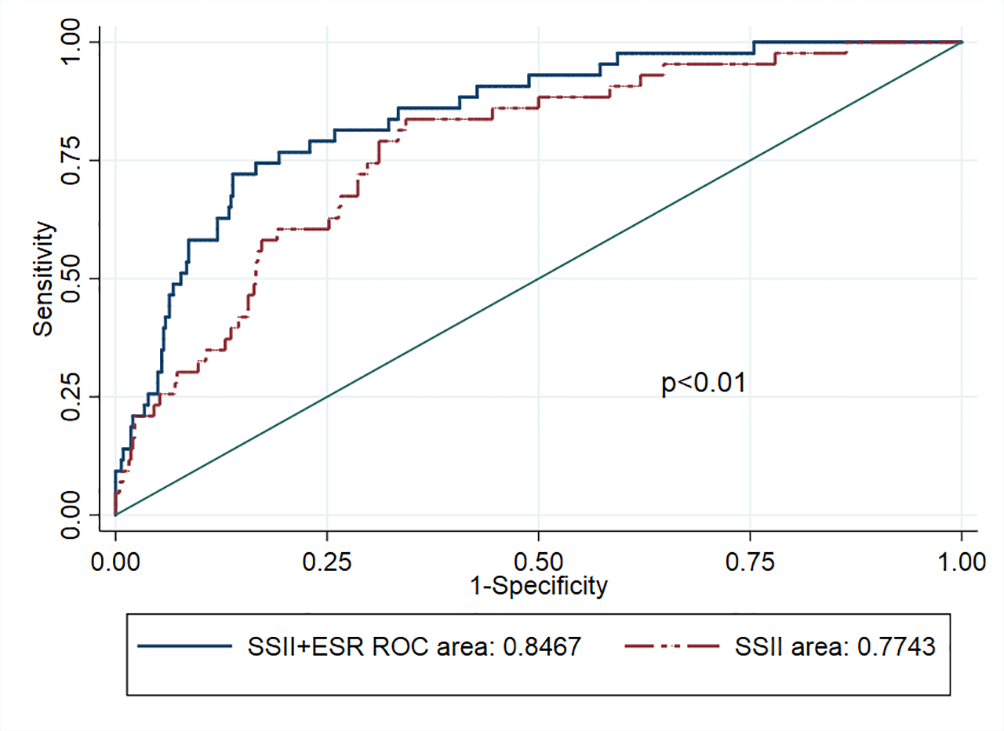

Supplement: Supplementary file 3 — Additional file 3: Supplementary Fig. 2. Receiver operating characteristic (ROC) curve for the combined models and SYNTAX score II (SSII) alone in predicting acute heart failure. [file 12872_2020_1490_MOESM3_ESM.tif]
